# Gene expression underlying enhanced, steroid-dependent auditory sensitivity of hair cell epithelium in a vocal fish

**DOI:** 10.1186/s12864-015-1940-3

**Published:** 2015-10-14

**Authors:** Daniel J. Fergus, Ni Y. Feng, Andrew H. Bass

**Affiliations:** Department of Neurobiology and Behavior, Cornell University, Ithaca, NY 14853 USA; Current Address: North Carolina Museum of Natural Sciences, Genomics and Microbiology, Raleigh, NC 27601 USA

**Keywords:** Hearing, Hair cells, Saccule, Transcriptome, Frequency sensitivity, Ion channels, Steroid hormones

## Abstract

**Background:**

Successful animal communication depends on a receiver’s ability to detect a sender’s signal. Exemplars of adaptive sender-receiver coupling include acoustic communication, often important in the context of seasonal reproduction. During the reproductive summer season, both male and female midshipman fish (*Porichthys notatus*) exhibit similar increases in the steroid-dependent frequency sensitivity of the saccule, the main auditory division of the inner ear. This form of auditory plasticity enhances detection of the higher frequency components of the multi-harmonic, long-duration advertisement calls produced repetitively by males during summer nights of peak vocal and spawning activity. The molecular basis of this seasonal auditory plasticity has not been fully resolved. Here, we utilize an unbiased transcriptomic RNA sequencing approach to identify differentially expressed transcripts within the saccule’s hair cell epithelium of reproductive summer and non-reproductive winter fish.

**Results:**

We assembled 74,027 unique transcripts from our saccular epithelial sequence reads. Of these, 6.4 % and 3.0 % were upregulated in the reproductive and non-reproductive saccular epithelium, respectively. Gene ontology (GO) term enrichment analyses of the differentially expressed transcripts showed that the reproductive saccular epithelium was transcriptionally, translationally, and metabolically more active than the non-reproductive epithelium. Furthermore, the expression of a specific suite of candidate genes, including ion channels and components of steroid-signaling pathways, was upregulated in the reproductive compared to the non-reproductive saccular epithelium. We found reported auditory functions for 14 candidate genes upregulated in the reproductive midshipman saccular epithelium, 8 of which are enriched in mouse hair cells, validating their hair cell-specific functions across vertebrates.

**Conclusions:**

We identified a suite of differentially expressed genes belonging to neurotransmission and steroid-signaling pathways, consistent with previous work showing the importance of these characters in regulating hair cell auditory sensitivity in midshipman fish and, more broadly, vertebrates. The results were also consistent with auditory hair cells being generally more physiologically active when animals are in a reproductive state, a time of enhanced sensory-motor coupling between the auditory periphery and the upper harmonics of vocalizations. Together with several new candidate genes, our results identify discrete patterns of gene expression linked to frequency- and steroid-dependent plasticity of hair cell auditory sensitivity.

**Electronic supplementary material:**

The online version of this article (doi:10.1186/s12864-015-1940-3) contains supplementary material, which is available to authorized users.

## Background

Understanding how genes are regulated within neural networks to produce and modify behavior is a major goal in neuroscience and behavioral genetics. One strategy for achieving this objective is to use model systems for identifying changing patterns of gene expression under different behavioral states. Exemplars include circadian rhythms in flies and mice e.g., [1–4], alternative foraging and aggressive behavioral states of honey bees e.g., [5, 6], and vocal-acoustic systems of songbirds e.g., [7]. Here, we used transcriptome analyses to investigate the genetic underpinnings of reproductive state- and steroid-dependent plasticity in auditory sensitivity of a teleost fish, the midshipman.

Midshipman hearing is an excellent model of neural plasticity for several reasons. First, midshipman exhibit reproductive state-dependent behavioral responses to playback of advertisement calls [8]. Second, these behavioral changes are paralleled by concurrent changes in peripheral auditory sensitivity, both at the level of hair cells and eighth nerve, ganglion cell afferents, especially for the advertisement call's upper harmonics [9–11]. Third, auditory hair cell plasticity can be explained, in part, by changes in the abundance of ion channels that underlie frequency tuning [12]. Fourth, reproductive state-dependent variation in eighth nerve encoding of frequency is steroid-dependent [13], providing a model for steroid-sensitive hearing variation in humans, in which age-related auditory deficits in post-menopausal woman can be ameliorated with estrogen therapy [14]. Fifth, the ease of collecting and housing midshipman fish in captivity facilitates downstream testing of identified candidate genes in a wild population of vertebrates.

To date, we have successfully employed hypothesis-driven approaches to identify neuro-molecular mechanisms of seasonal variation in peripheral auditory function, namely in the hair cell epithelium and eighth nerve afferents of the saccule, the main auditory division of the inner ear in many teleost fish including midshipman [8, 15]. This has included examining the function, location, and abundance of ion channels and steroid receptors in the auditory periphery [12, 13, 16, 17]. Neurophysiological studies show that either estrogen or testosterone can transform the frequency sensitivity of the saccular afferents of non-reproductive animals to that of summer animals [13]. Aromatase (estrogen synthase) and estrogen receptors are expressed within ganglion cells and the hair cell saccular epithelium, respectively [16, 17], indicating that steroids can act directly within the peripheral auditory system. Neurophysiology combined with pharmacology, quantitative reverse-transcriptase PCR, and immunohistochemistry also indicates that large-conductance potassium (BK) channel expression in saccular hair cells plays a key role in regulating the observed seasonal plasticity in auditory sensitivity [12].

Though our hypothesis-driven approach has been fruitful, it provides a limited view of the cascade of events underlying steroid-dependent, seasonal auditory plasticity. Advances in next-generation sequencing and high-throughput analyses can provide a global view of gene expression. Here, we use transcriptome sequencing to uncover seasonal and reproductive state-dependent differences in transcript abundances within the auditory saccular epithelium. We identified a suite of candidate genes and pathways with known auditory function in midshipman fish, and vertebrates in general, that likely underlie seasonal and reproductive state-dependent variation in hearing. The results have been reported, in part, in abstract form [18].

## Results and discussion

### Transcriptome characterization

Midshipman have two male reproductive morphs; we used type I males here because they are the most abundant during collections and have the most dynamic vocal repertoire [19, 20]. Given the lack of sex [11] and male morph [21] differences in auditory hair cell physiology, the use of only type I males should not impact our results. Here, we focus on the subset of transcriptome sequences from the auditory saccular epithelium (SE) of reproductive (summer) and non-reproductive (winter) type I males that were previously used for physiological examination of auditory sensitivity [11].

We sequenced and annotated transcriptome libraries produced from the SE, along with libraries derived from the vocal motor nuclei (VMN) and the hindbrain region surrounding the VMN. The VMN and hindbrain samples were used for a comprehensive companion study that identified daily and seasonal variation in gene expression patterns in the VMN, the final node of the vocal control network that sends a command signal to the vocal muscles [22]. In that study, the RNA-seq determined expression patterns of 28 genes were validated using quantitative PCR, showing a strong correlation between the two measures of transcript abundance [22].

**Fig. 1:**
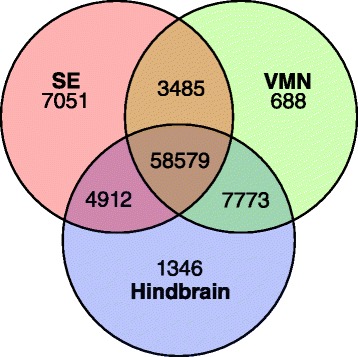
Common and unique transcripts among tissues. The Venn diagram illustrates the common and unique transcripts across saccular hair cell epithelium (SE), VMN, and hindbrain tissues [22]. Most transcripts were shared among all tissues, while SE had the largest number of unique transcripts, consistent with their unique (epithelial) tissue type

We obtained approximately 200 million total paired-end reads; over 20 million reads were produced from each pooled SE sample (Table 1). Using the Trinity software package (version r2013-02-15 [23, 24]) we assembled all the reads together into a final set of 83,967 unique transcripts (isoforms) after filtering for quality of reads and of assembled transcripts [22]. The final transcriptome assembly, reported in our companion study [22], had a mean length of 1713.57 ± 1585.21 bp (N50 = 2647) with 40,656 genes (components) across brain and SE samples. The assembled transcriptome is available on the NCBI Transcriptome Shotgun Assembly and Sequence Read Archive databases under BioProject accession number PRJNA269550. Using Blast2GO, we found significant annotation hits for 74,000 (88 %) of our assembled transcripts, with most top blast hits being to teleost fish (see [22]). Mapping individual reads back to the assembled transcriptome and comparing FPKM (fragments per kilobase per million reads) values showed that most transcripts were expressed (FPKM > 0) in all examined tissue types, though the SE possessed the largest number of tissue-specific transcripts that had no expression (FPKM = 0) in either VMN or hindbrain (Fig. 1). This likely reflects the different developmental trajectory and cell types of the inner ear (placode) relative to the VMN and hindbrain (rhombomeres) (see [25]).

**Table 1:** Number of saccular epithelium reads by reproductive state before and after quality filtering

Reproductive state	Raw forward reads	Filtered paired-end reads
Reproductive	23112842	21401113
Non-reproductive	24208024	21617526

### Reproductive-state specific expression

We first examined top expressed transcripts and pathways in reproductive and non-reproductive SE, regardless of differential expression. For transcripts with significant Blast2GO annotations, 6 of the top 10 most highly expressed transcript annotations were common to both reproductive and non-reproductive SE although they varied in expression levels (Table 2). Four of the top 10 most abundant transcripts in the reproductive SE encode ribosomal proteins, while only one of those in the non-reproductive SE encodes a ribosomal protein. The translationally controlled tumor protein (*tpt1*), which functions to prevent cell death [26, 27], was among the most abundant transcripts in the reproductive SE and may play a role in the increased hair cell numbers previously reported in the reproductive SE [28].

**Table 2:** Top 10 most highly expressed annotated saccular epithelium transcripts

Reproductive	Sequence ID	FPKM	Non-reproductive	Sequence ID	FPKM
Myoglobin	comp194478_c0_seq1	25048	Serine threonine-protein kinase samkc-like isoform x2	comp203376_c0_seq1	36792
Serine threonine-protein kinase samkc-like isoform x2	comp203376_c0_seq1	24585	Myoglobin	comp194478_c0_seq1	29279
Mucin-22-like	comp203306_c0_seq3	9686	Hemoglobin subunit beta-like	comp194456_c0_seq1	21830
Male-specific protein	comp237277_c0_seq1	8890	Mucin-22-like	comp203306_c0_seq3	15462
Inner ear-specific collagen-like	comp221658_c0_seq1	8616	Inner ear-specific collagen-like	comp221658_c0_seq1	13183
Translationally controlled tumor protein	comp126388_c0_seq1	7895	Male-specific protein	comp237277_c0_seq1	9766
Ribosomal protein l12	comp172912_c0_seq1	6996	Matrilin-4 isoform 1	comp210154_c1_seq1	8186
40S ribosomal protein s27	comp194407_c0_seq1	6806	40S ribosomal protein s8	comp126398_c0_seq1	7949
40S ribosomal protein s8	comp126398_c0_seq1	6681	β-actin	comp126442_c0_seq1	7226
60s ribosomal protein l32	comp126412_c0_seq1	6011	α-type globin	comp221632_c0_seq3	6652

We also identified the top 10 KEGG (Kyoto Encyclopedia of Genes and Genomes) pathways based on the number of annotated transcripts that mapped to each pathway for reproductive and non-reproductive SE (Table 3). It is noteworthy that each of the top KEGG pathways for the reproductive SE are represented by far more transcripts, on average, than the top non-reproductive KEGG pathways. This may have resulted, in part, from completely "turning off" more complex pathways in the non-reproductive SE and/or decreased transcription of certain genes. For example, among the highly represented KEGG pathways in reproductive SE were processes involved in cellular respiration, including oxidative phosphorylation, glycolysis, TCA cycle, and pyruvate metabolism. While cellular respiration unquestionably occurs in the SE throughout the entire year to support year-round hearing [10, 11], the reduced transcript representation of these cellular respiration KEGG pathways in non-reproductive SE suggests a higher energetic demand in the reproductive state that corresponds to greater SE auditory sensitivity [9, 11].

**Table 3:** Top 10 KEGG pathways in the saccular epithelium by number of transcripts

Reproductive	KEGG ID	Transcripts	Non-reproductive	KEGG ID	Transcripts
Purine metabolism	map00230	107	Purine metabolism	map00230	66
Oxidative phosphorylation	map00190	50	Pyrimidine metabolism	map00240	27
Glycolysis/Gluconeogenesis	map00010	45	Thiamine metabolism	map00730	13
Pyrimidine metabolism	map00240	38	Phosphatidylinositol signaling system	map04070	12
Citrate cycle (TCA cycle)	map00020	30	Aminoacyl-tRNA biosynthesis	map00970	10
Carbon fixation pathways in prokaryotes	map00710	28	Lysine degradation	map00310	9
Pyruvate metabolism	map00620	25	Glycerophospholipid metabolism	map00564	7
Glutathione metabolism	map00480	25	Inositol phosphate metabolism	map00562	7
Carbon fixation in photosynthetic organisms	map00710	24	Various types of N-glycan biosynthesis	map00513	6
Glycine, serine and threonine metabolism	map00260	24	One carbon pool by folate	map00670	6

**Fig. 2:**
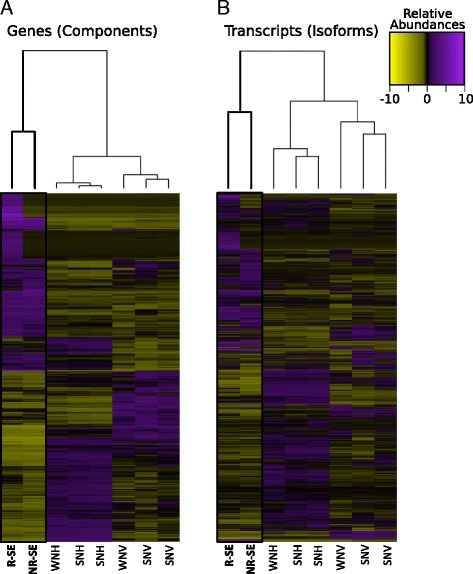
Heatmaps of tissue specific expression. Heatmaps showing normalized expression of differentially expressed (**a**) genes and (**b**) transcripts among saccular epithelium (SE) and brain tissues. Differential expression is based on a minimum 4-fold differential variation among tissues with a maximum false discovery rate (FDR) of 0.001. While samples grouped by tissue type, the SE showed strong differentiation by season. The SE columns, the focus of this study, are highlighted by a black box. Abbreviations: R-SE, reproductive saccular epithelium; NR-SE, non-reproductive saccular epithelium; WNH, winter night hindbrain; SMH, summer morning hindbrain; SNH, summer night hindbrain; WNV, winter night VMN (vocal motor nucleus); SMV, summer morning VMN; SNV, summer night VMN

**Fig. 3:**
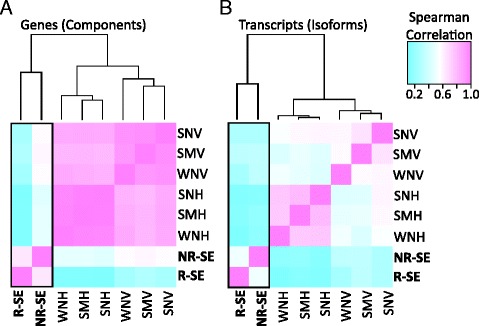
Spearman's correlation of gene and transcript expression. Sample relationships based on **(a)** gene and **(b)** transcript expression were scaled to a color gradient based on Spearman's correlations among tissue groups. As in Fig. 2, the saccular epithelium (SE) tissues grouped together, but still showed strong differentiation between seasons. This difference is much more apparent at the level of the transcripts than the genes. The SE columns, the focus of this study, are highlighted by a black box. Abbreviations are as in Fig. 2

We examined the differential regulation of gene expression in the SE across reproductive states by performing differential expression analyses of genes (components) and transcripts (isoforms). We compared transcript abundances across all the SE, VMN and surrounding hindbrain tissue groups using a false discovery rate (FDR) threshold of 0.001 and a minimum four-fold differential abundance cutoff (Figs. 2, 3). Based on pairwise comparisons of differential transcript abundances, the samples were more similar between time points for a single tissue type than between tissues. Furthermore, in all tissue types, transcript isoforms showed greater differential expression than gene components across time points, suggesting that variation across seasons was determined more by differential splicing than by turning the expression of a given gene on or off. The substantial expression change parallels the observed neurophysiological change in SE auditory sensitivity between reproductive and non-reproductive individuals [9, 11]. Furthermore, the SE showed higher seasonal differentiation in both gene and transcript expression relative to either VMN or the surrounding hindbrain (Figs. 2, 3), suggesting that at the level of hair cells and motoneurons, seasonal variation in hearing sensitivity requires greater transcriptional changes than the vocal motor system.

Focusing specifically on transcripts differentially expressed between reproductive and non-reproductive SE, we identified 4713 and 2221 upregulated in reproductive and non-reproductive SE, respectively. Performing the same analysis with genes rather than transcripts produced 878 and 24 genes upregulated in reproductive and non-reproductive SE, respectively. We employed a Fisher's Test for gene ontology (GO) term enrichment with Blast2GO to determine whether there was an overrepresentation of any classes of functionally similar transcripts that were differentially regulated across seasons. Many GO terms were over-represented among transcripts upregulated in reproductive SE, while none were over-represented among transcripts upregulated in non-reproductive SE (Table 4). The most significantly enriched GO terms were primarily related to translation (e.g., ribosomal proteins) and cellular respiration, supporting results of the overall most abundant transcripts (Table 2) and most supported KEGG pathways (Table 3) discussed above.

**Table 4:** Enriched GO terms among upregulated transcripts in reproductive saccular epithelium

	GO-ID	GO term	*p*
Biological process	GO:0000184	Nuclear-transcribed mRNA catabolic process, nonsense-mediated decay	1.2E-14
	GO:0006614	SRP-dependent cotranslational protein targeting to membrane	6.6E-14
	GO:0006415	Translational termination	2.4E-13
	GO:0015986	ATP synthesis coupled proton transport	3.9E-12
	GO:0019083	Viral transcription	6.7E-11
	GO:0006446	Regulation of translational initiation	9.7E-11
	GO:0006120	Mitochondrial electron transport, NADH to ubiquinone	8.6E-10
	GO:0006744	Ubiquinone biosynthetic process	2.1E-09
	GO:0006123	Mitochondrial electron transport, cytochrome c to oxygen	2.1E-09
	GO:0006096	Glycolysis	1.2E-08
	GO:0006457	Protein folding	1.7E-08
	GO:0006094	Gluconeogenesis	2.6E-08
	GO:0000028	Ribosomal small subunit assembly	2.0E-07
	GO:0031101	Fin regeneration	2.1E-07
	GO:0006448	Regulation of translational elongation	2.1E-07
	GO:0019643	Reductive tricarboxylic acid cycle	1.6E-06
	GO:0015976	Carbon utilization	3.0E-05
	GO:0030036	Actin cytoskeleton organization	3.4E-05
	GO:0006364	rRNA processing	3.9E-05
	GO:0005980	Glycogen catabolic process	6.2E-05
Molecular function	GO:0003735	Structural constituent of ribosome	4.6E-37
	GO:0008137	NADH dehydrogenase (ubiquinone) activity	6.2E-11
	GO:0003743	Translation initiation factor activity	1.1E-09
	GO:0004129	Cytochrome-c oxidase activity	2.5E-09
	GO:0051082	Unfolded protein binding	9.4E-08
	GO:0046933	Proton-transporting ATP synthase activity, rotational mechanism	5.7E-07
	GO:0003746	Translation elongation factor activity	2.4E-06
	GO:0019843	rRNA binding	1.8E-05
	GO:0004365	GAPDH (NAD+) (phosphorylating) activity	6.7E-05
	GO:0072542	Protein phosphatase activator activity	8.5E-05
	GO:0016936	Galactoside binding	9.9E-05
Cellular component	GO:0022627	Cytosolic small ribosomal subunit	1.5E-22
	GO:0022625	Cytosolic large ribosomal subunit	1.3E-12
	GO:0005852	Eukaryotic translation initiation factor 3 complex	1.6E-10
	GO:0045277	Respiratory chain complex IV	2.1E-09
	GO:0045095	Keratin filament	6.0E-09
	GO:0005747	Mitochondrial respiratory chain complex I	2.0E-08
	GO:0045261	Proton-transporting ATP synthase complex, catalytic core F(1)	3.5E-07
	GO:0042470	Melanosome	4.3E-05
	GO:0005730	Nucleolus	5.7E-05
	GO:0005861	Troponin complex	5.9E-05
	GO:0005832	Chaperonin-containing T-complex	6.2E-05
	GO:0000276	Mitochondrial H^+^-transporting ATP synthase complex, coupling factor F(o)	6.2E-05

Enrichment was based on a one-tailed Fisher's exact test with *p*-values < 10^−5^. Transcripts involved in translation and cellular respiration were notably upregulated in the reproductive saccular epithelium. No GO terms were enriched among non-reproductive, upregulated transcripts

As a control for our differential analyses in the SE, we performed comparable seasonal analyses for sample groups of the hindbrain region surrounding VMN that were used in our companion study of the vocal motor system [22]. Using the same differential expression criteria, we found that 2157 and 1984 transcripts were upregulated in reproductive and non-reproductive hindbrains, respectively. There were 3 and 12 genes upregulated in the reproductive and non-reproductive hindbrain, respectively. Among the differentially expressed transcripts, only a single GO term, “protein-binding”, was significantly enriched in the reproductive hindbrain. Thus, transcript abundance and GO term enrichment differences did not reflect ubiquitous seasonal or reproductive state variation, but rather SE-specific transcriptional regulation.

Taken together, the results identified broad changes in gene expression between reproductive and non-reproductive states of high and low saccular activity, respectively. The substantially greater number of transcripts and genes upregulated in reproductive relative to non-reproductive samples suggested a general increase in transcriptional activity. The absence of this pattern in the hindbrain region surrounding VMN indicated that the increased transcription in the reproductive state is an SE-specific phenomenon. Furthermore, the enriched GO terms (Table 4) of the differentially expressed transcripts pointed to a much greater translational and metabolic activity in the reproductive SE than the non-reproductive SE, consistent with the most abundant annotations and KEGG pathways reported in Tables 2 and 3. We thus conclude that the SE is transcriptionally, translationally, and metabolically more active in reproductive than non-reproductive individuals.

### Candidate gene identification

The overarching goal of this study was to identify candidate genes that may influence seasonal variation in auditory frequency sensitivity. While the analyses above suggested broad changes in activity and metabolism across reproductive states, we hypothesized that the known physiological changes in auditory sensitivity likely depend predominantly on changes in a smaller subset of genes important for neural function and neuronal excitability e.g., [12]. While our approach here may have missed variation of low abundance transcripts and small magnitude expression differences, we identified numerous candidate genes for further study. Furthermore, many of the identified candidates have previously been implicated in vertebrate auditory function (Table 5, [29]).

**Table 5:** Differentially expressed candidate genes in the saccular epithelium with reported auditory functions and comparison to mouse hair cell expression

Top blast hit description	Season	Citation	Mouse HC
Vesicular glutamate transporter 3	R	[81]	+
Estrogen-related receptor β type 1	R	[33]	=
Thyroid hormone receptor α	R/NR	[82]	=
Connexin 43 (Gap junction α-1)	R/NR	[83]	-
Neuronal acetylcholine receptor subunit α-9-ii^a^	R	[36]	+
Potassium voltage-gated channel subfamily a member 10	R	[84]	+
Sodium channel protein type 8 subunit α	R	[85]	=
Diaphanous homolog 1-like isoform x2	R	[42]	=^b^
Insulin gene enhancer protein isl-1	R	[41]	+
Estrogen-related receptor γ	R/NR	[35]	+
Calcium-activated potassium channel subunit α-1 (BK)	R	[12]	=
Connexin 30 (Gap junction β-6)	R/NR	[65]	-
Trimeric intracellular cation channel type a	R	[86]	+
Voltage-dependent calcium channel subunit α-2 δ-3	R/NR	[87]	=

These differential expressed transcripts have previously been implicated in peripheral auditory function. Shown are the top Blast hit descriptions, the season during which transcript abundance was highest (R: reproductive; NR: non-reproductive), and a citation for the auditory role of that gene. In cases where both seasons are listed, different isoforms were upregulated in both seasons. The "Mouse HC" column represents comparisons of the average normalized mRNA transcript abundances of FAC sorted hair cells to that of surrounding cells from embryonic day 16 and postnatal days 0, 4, and 7 mouse cochlea [29]. We indicated whether expression in hair cells was not substantially different (=), >2 fold higher (+), or >2 fold lower (−), than in the surrounding cells of the cochlea. The first 8 transcripts in this table were further supported by an examination of postnatal day 1 mouse organ of corti [88]. ^a^Neuronal acetylcholine receptor subunit α-9-ii (Chrna9-ii) is not present in mammals; comparable genes Chrna9 and Chrna10 transcripts are both more abundant in hair cells than surrounding cells. ^b^Diaphanous homolog 1 is not reported in [29] and this comparison is based only on [88]

Candidate genes upregulated in the reproductive SE that support prior studies of midshipman auditory function included *estrogen related receptors* (ERR), *neuronal acetylcholine receptor subunit α-9-ii*, *insulin gene enhancer protein isl-1, and diaphanous 1*. The ability of estrogen to enhance auditory sensitivity in the midshipman SE is well established [13], and estrogen receptors have been localized to the SE [16]. ERRs exert estrogen-like actions by activating genes regulated by estrogen-response elements in the absence of the ligand [30–32], and mutations of ERRs have been implicated in hearing impairments in mice and humans ([33–35]). Increased ERR expression may serve as a supplemental or alternative means to estrogenic actions in regulating genes important for maintaining high-frequency auditory sensitivity.

The *neuronal acetylcholine receptor subunit α-9-ii*, also upregulated in reproductive SE, is expressed in hindbrain neurons that directly innervate the inner ear (i.e., auditory efferents) of teleosts [36] that have been shown in midshipman to be part of a vocal corollary discharge pathway linking the hindbrain vocal pattern generator to the auditory saccule [37]. In mammals, which lack the α-9-ii receptor, acetylcholine receptor subunits α-9 and α-10 are highly expressed in auditory hair cells [29] and form heterotetramers at the auditory efferent synapses with cochlear hair cells (see [38]), likely regulating the dynamic range of hearing and protecting against environmental or self-generated acoustic trauma. In midshipman, acetylcholine receptor subunit α-9-ii may either be involved in setting the auditory sensitivity thresholds across frequencies or help protect the SE in the potentially more acoustically active tidal environment inhabited during the reproductive summer compared to the deep water environment inhabited during the non-reproductive winter. Ambient sound levels during the summer reproductive period may be higher due to increased vocal activity especially in and close to nests [19, 20, 39], as well as increased noise levels in the shallow water environment e.g., [39, 40] where midshipman build nests and spawn [19, 20].

The *insulin gene enhancer protein isl-1* is an interesting candidate gene based on recent work by Huang et al. [41], which showed that over-expression of *Isl-1* in mouse hair cells prevented age-related and noise-induced hearing loss resulting from hair cell apoptosis or neural degeneration. Another candidate gene with similar implications for auditory sensitivity and hair cell proliferation is *diaphanous 1* whose mutation contributes to progressive hearing loss [42]. Products of the *diaphanous* genes contribute to cytoskeletal function including establishing cell polarity and shape [43–45]. Both of these candidate genes could contribute to the increased auditory sensitivity at high frequencies as well as the reported increase in hair cell number in the reproductive SE [28].

There was little overlap between the candidate genes identified in this study and those that are activated during regeneration following acoustic trauma in zebrafish [46]. Given the seasonal variation in hair cell abundance in midshipman [28], we might expect some overlap among these processes. The presence of some similar classes of genes, such as myosin genes and orthologous nuclear receptors, in both analyses may stem from the regeneration of new hair cells in both model systems. However, the amount of hair cell regeneration and the underlying mechanisms may vary substantially between trauma-induced and naturally occurring seasonal regeneration.

There were additional upregulated genes in the reproductive SE that have not previously been directly implicated in auditory function but still serve as important candidates for consideration and future validation. These included steroid related genes such as the transcript *hydroxysteroid 11-β-dehydrogenase 1*, which converts cortisol to the inactive metabolite cortisone (see [47, 48]), as well as transcripts encoding glucocorticoid receptors (Additional file 1). Though cortisol has not been directly implicated in auditory function, there is extensive evidence for a role of glucocorticoids in hearing [49–53] and in lateral line hair cell regeneration [54]. In teleost fish, *hydroxysteroid 11-β-dehydrogenase* also converts 11-beta-hydroxytestosterone to 11-ketotestosterone (11KT), a non-aromatizable androgen detectable only in reproductive type I males [55, 56]. While 11KT has an effect on vocalization [57, 58], its role in auditory physiology has not been examined. The auditory sensitivity of primary afferents innervating the SE of non-reproductive fish can be shifted to that of reproductive fish by testosterone [13], which may act via local conversion to estrogen by aromatase in the ganglion cells [17]. This mechanism would compete with the conversion of testosterone to 11KT, which could only act by direct activation of an androgen receptor (AR). In situ hybridization shows ARβ mRNA in the region directly adjacent to saccular hair cells [59]. Transcripts of both ARα and ARβ are detectable by qPCR at approximately equal abundances in the SE of all reproductive morphs (D. Fergus and A. Bass, unpublished observations), but seasonal variation in expression of either AR has not been directly tested and was not detected in our results here. Many genes like *hydroxysteroid 11-β-dehydrogenase 1* could be critical for the physiological changes across reproductive states in midshipman SE, but have not been studied in the context of auditory plasticity prior to this transcriptome differential expression analysis.

Neurophysiology shows that ion channels and steroid hormones are critical to the increased auditory sensitivity in reproductive midshipman fish [12, 13] (see also [60] for qPCR of steroid receptors in SE of a cichlid fish). To take a more targeted approach for our candidate gene search, we identified 1547 ion channel transcripts and 361 steroid-related transcripts within our entire assembled transcriptome and performed differential expression analyses with each of these transcript subsets. This approach reduced the number of pairwise comparisons, allowing us to potentially increase our sensitivity to detect differentially expressed transcripts with functional importance. As with the whole transcriptome, substantially more steroid-related (Additional file 1) and ion channel (Additional file 2) transcripts were upregulated in reproductive compared to non-reproductive SE.

Among the ion channels upregulated in reproductive SE were large conductance, calcium-activated potassium (BK) channel transcripts (Table 5, Additional file 2) that have been localized to saccular hair cells in midshipman, shown to vary in abundance across seasons and to regulate auditory sensitivity in midshipman adults and zebrafish larvae [12, 61]. Numerous other potassium channel transcripts were also differentially regulated across seasons (Additional file 2), which may be necessary for the fine-tuning of auditory thresholds, as demonstrated in other vertebrates [62] and suggested by our auditory physiology studies of the SE [12].

One steroid-related and several channel transcripts identified as upregulated in reproductive SE were also upregulated in the hindbrain and/or VMN of reproductive state fish (see companion RNA-seq study [22]) (Table 6). The common reproductive-state dependent regulation of these transcripts may support broadly shared motor and sensory functions. Two channel transcripts that showed reproductive upregulation in the VMN and SE, but not the hindbrain, *connexin 30* (*Cx30*, *gap junction* β*-6*) and *calcium-activated potassium channel subunit α -1* (*BK*) (Table 6), are particularly interesting in light of our previous neurophysiological examinations of midshipman vocal and auditory systems. Connexins are gap junction proteins that contribute to electrical coupling between cells and are abundant in glia [63]. Though not yet tested, *Cx30* might support the known electrical coupling between VMN motoneurons and, in turn, the extreme, population level synchronicity observed for VMN [64]. In the inner ear, Cx30 occurs between supporting cells in the cochlear hair cell epithelium; mice lacking Cx30 show severe hearing loss [65]. There is evidence for gap junctions between supporting cells of the SE and possibly between hair cells and supporting cells in toadfish from the same family as midshipman [66]. BK channels, as noted earlier, are more abundant in the SE of reproductive midshipman, playing a prominent role in the sensitivity of SE hair cells to the full ~100-400 Hz spectral range of their vocalizations [12]. BK channels may also contribute to high fidelity firing in VMN that codes for vocalization pulse repetition rates and fundamental frequencies of ~100-110 Hz [20]. In support of this potential vocal function, recent studies demonstrate a role for BK channels in high fidelity firing (~50-100 Hz) by Purkinje cell axons in the cerebellum [67]. The co-regulation of such genes in two highly divergent neural systems, one sensory and one motor, is compelling given the importance of sender-receiver/vocal-auditory coupling in the acoustic communication system of the plainfin midshipman [8, 12, 13, 37, 68]**.**

**Table 6:** Channel and steroid-related candidate genes upregulated in the vocal system

Reproductive	Vocal Upregulation
Cholesterol 25-hydroxylase protein member 1	H/VMN
Connexin 43 (Gap junction α-1)	H/VMN
Voltage-gated potassium channel subfamily c member 4	H/VMN
Sodium channel protein type 8 subunit α	H/VMN
Two pore calcium channel protein 1	H/VMN
Connexin 30 (Gap junction β-6)	VMN
Calcium-activated potassium channel subunit α-1 (BK)	Night VMN
Transient receptor potential cation channel subfamily m member 7	VMN
Anoctamin-10	Night VMN

Transcripts of these candidate genes, upregulated in reproductive SE, were also more abundant in vocal regions of the CNS of reproductive type I males [22]. Some transcripts were upregulated throughout the hindbrain and VMN (H/VMN), some were upregulated only in the VMN (VMN), and others had increased abundance restricted to VMN at night (Night VMN), the time of peak vocal activity

## Conclusion

We uncovered the molecular underpinnings of reproductive state-dependent variation of auditory sensitivity in midshipman fish. Our results suggested broad changes in transcriptional, translational, and metabolic activity occurring in the SE across reproductive states, with higher activity in the reproductive state. In addition to these broad changes, differential expression analyses identified a number of potential candidate genes underlying seasonal changes in auditory physiology. Some of these genes, such as potassium channels and steroid biogenesis enzymes, are highly consistent with our previous work, while others, like *insulin gene enhancer protein isl-1*, *neuronal acetylcholine receptor α-9-ii* and *diaphanous 1*, are implicated in mammalian hair cell function and thus provide novel targets for future investigation in fish model systems.

We have previously shown that the magnitude of the seasonal change in hearing thresholds in the SE is significantly greater for encoding the higher frequency, upper harmonics of the male advertisement call ([11], also see [13]). Midshipman fish migrate from nest sites in the shallow intertidal zone to deep off shore sites during the non-reproductive winter season e.g. [56]. Despite the apparent attenuation of transcription, translation, and metabolic activity in the non-reproductive SE that we report here, the SE retains robust sensitivity to low frequency sound (≤100 Hz) [10–13, 68]. Such low frequency sensitivity in deep water sites has been proposed to be important for detection of the brief (~200 msec), low frequency agonistic grunts of conspecifics that are produced all year long [19, 20] and the low frequency calls of marine mammals, both of which will have a greater transmission distance in deeper water (see [39, 69]). Seasonal changes in hearing are not observed in a closely related species of toadfish that does not migrate to deeper waters during the non-reproductive season [70]. Perhaps the basal condition for toadfishes is high sensitivity across a wide range of frequencies and spectral peaks. In this case, selective pressure may have actively suppressed sensitivity to high frequencies while maintaining sensitivity for low frequencies in the non-reproductive winter SE of midshipman fish, rather than actively enhancing the higher frequency hearing in a summer reproductive fish. While this does not drastically change our questions regarding seasonal variation in auditory sensitivity, it can inform our thinking about how and why species like midshipman evolved the physiological and genetic mechanisms underlying frequency-dependent seasonal plasticity in hearing.

## Methods

### Collection

All fish used in this study were type I males. The SE were from reproductive males collected in summer and non-reproductive males collected in winters of 2009 and 2010 in California and Washington. These fish, previously used to examine seasonal variation in auditory sensitivity and steroid levels [11], were collected from nest sites, shipped back to Cornell University, and housed in artificial seawater aquaria maintained at 16 °C until they were used for neurophysiology and sacrificed to collect tissues. The ears were removed, dissected to isolate the SE from surrounding ear tissue, immediately frozen in liquid nitrogen, and stored at -80 °C until being used for RNA isolation. All procedures used here were approved by Cornell University's Institutional Animal Care and Use Committee.

### Library construction

The methods for library construction, sequencing, and transcriptome assembly here are the same as those used in our companion study of the vocal network [22]. Total RNA was isolated from the SE of 6 reproductive and 6 non-reproductive fish using the Trizol reagent (Invitrogen) following the manufacturer's standard protocol. The isolated total RNA was quantified using the Qubit RNA HS quantification kit (Invitrogen) and equal quantities of RNA from each ear were pooled by reproductive state. DNase I (Ambion) treatment was performed on each pool to remove contaminating DNA.

We constructed indexed, strand-specific cDNA libraries using the deoxyuridine triphosphate (dUTP)/uracil-DNA glycosylase (UDG) approach described by Zhong et al. [71]. Briefly, mRNA was purified from the total RNA using Dynabeads Oligo(dT)_25_ (Life Technologies) and then fragmented to approximately 200 bp with divalent cation buffer (SuperScript III buffer, Life Technologies). This fragmented mRNA was used to produce first-strand cDNA with dNTPs and SuperScript III enzyme. We then produced second-strand cDNA with dUTP substituted for dTTP, dA-tailed the double-stranded DNA fragments, and ligated Y-adapters created by annealing primers with both complimentary and non-complimentary regions.

After the Y-adapters had been ligated to the double-stranded DNA fragments, we purified and size selected them using AMPure beads (Beckman Coulter, Inc.). The uracil containing second-strand DNA was digested with uracil-DNA glycosylase. After digesting the second-strand, the remaining first-strand cDNA had unique sequence tags on the 5' and 3' ends that served as priming sites for PCR primers containing indexes and epitopes necessary for Illumina sequencing. We used 14 cycles of PCR reactions to produce double-stranded cDNA fragments that were uniquely indexed for summer reproductive and winter non-reproductive SE. The resulting DNA was purified, verified by both gel electrophoresis and Agilent Bioanalyzer, which showed peaks between 251 and 257 bp. The DNA was quantified with the Qubit dsDNA HS quantification kit (Invitrogen), and combined in equal quantities (20 ng per pool) with the vocal motor nucleus (VMN) and hindbrain libraries described in Feng et al. [22]. The 2000 ng multiplexed cDNA pool was 2×100 paired-end sequenced on the Illumina HiSeq2000 in the Cornell University Institute of Biotechnology Genomics Facility.

### Transcriptome assembly and annotation

The assembled and annotated transcriptome described here was the same transcriptome used for our companion study [22]. Illumina quality filtering was used to remove pairs of reads in which either of the paired reads was of poor quality. The Trimmomatic tool kit [72] was used to remove adaptor sequences and low quality nucleotides from the ends, and trimmed sequences of less than ten nucleotides were removed. Following filtering, there were 20.2 ± 2.4 million reads (mean ± SD) per tissue group remaining. These were transferred to the Pittsburgh Supercomputing Center's Blacklight system for *de novo* transcriptome assembly. Downstream analyses were performed with the Trinity version r2013-02-15 software package on Cornell’s Computational Biology Service Unit’s computers [23, 24, 73]. In examining previously identified genes in our initial assembly, we found several problematic contigs in which, for example, paralogous steroid receptors were assembled into single gene components or assembled transcripts contained long extraneous sequences on one end, apparently representing portions of transcripts from other genes. The observation of these chimeras is likely due to the whole genome duplication in teleosts (see [74, 75]) and our interest in genes with multiple known orthologs (eg., estrogen, androgen, and glucocorticoid receptors). To reduce such chimeric assemblies, we employed the jaccard_clip function as well as set the min_kmer_cov at 2. While these settings increased the likelihood of fragmentation of assembled transcripts, they substantially reduced the occurrence of chimeric transcripts in our final transcriptome and still maintained a final N50 value of 2647. These settings largely eliminated such chimeric assemblies among our closely examined transcripts. Following the initial assembly process, we filtered the transcriptome further to retain only transcripts that had an open reading frame (ORF) of at least 50 amino acids. While a 50 amino acid ORF is not stringent, we wanted to avoid eliminating potentially important short protein coding genes while reducing the number of non-coding genes. Though these non-coding genes may be critical to the variation in seasonal auditory physiology, their general lack of annotation makes such a bioinformatic analysis essentially impossible.

Using Trinity-supported downstream analysis tools, the initial HiSeq2000 reads were mapped back to the assembled transcriptome with Bowtie [76] in the RSEM [77] workflow to estimate abundances for each transcript and determine how well each assembled transcript was supported by the assigned reads. In cases of genes (components) with multiple transcripts (isoforms), if the number of reads that mapped to a given transcript (the IsoPct) was less than 1 % of the total number of reads that mapped to all the transcripts for that gene, we considered that transcript to be lowly supported and eliminated it from our final transcriptome assembly.

After assembling the reads and filtering out poorly supported transcripts, we annotated the full transcriptome based on similarity to sequences in the NCBI non-redundant protein database using Blast2GO [78]. Within Blast2GO, we used blastx to compare each transcript to the NCBI protein database with an e-value cutoff of 10^−10^. We performed subsequent blastn analyses for sequences without significant blastx hits, though this added few annotation due to our previously excluding transcripts with ORFs of less than 50 amino acids. We then used Blast2GO for mapping and annotation of the transcripts as well as performing InterProScan and GO-Enzyme Code assignments.

### Analysis of most abundant transcripts

We first performed analysis on the most highly expressed transcripts from reproductive and non-reproductive SE, regardless whether they were differentially expressed. In conjunction with analyses performed on differentially expressed transcripts below, this is useful for identifying reproductive-state dependent changes occurring at the level of the ear. Following Blast2GO annotation we identified the top 10 most abundant annotated transcripts within the reproductive and non-reproductive SE. We also mapped transcripts from reproductive and non-reproductive SE to KEGG functional pathways [79] using Blast2GO. The KEGG pathways were ranked in order of the number of transcripts assigned to each pathway in reproductive and non-reproductive SE, and the top 10 KEGG pathways were identified for each reproductive morph. This approach allowed us to compare the relative level of transcription dedicated to different pathways in the SE across reproductive states.

### Analysis of differentially expressed transcripts

Differential expression analyses were performed within Trinity using RSEM and edgeR [80] following Trinity's standard differential analysis protocol. Though we focused largely on differential transcript expression, we performed these differential expression analyses on both the genes and the transcripts to allow comparisons between regulation at the gene and isoform levels. We used edgeR to estimate the common dispersion using a subset of 228 core eukaryotic genes (CEGs) [89, 90] selected based on having little or no direct involvement in cellular respiration, transcription, or translation. Initial analyses indicated that transcripts with these functions are highly differentially expressed across reproductive states in the ears, and thus would likely give an inaccurate dispersion estimate. The calculated dispersion value of 0.13317 was used in determining differential expression. We used a minimum 4-fold abundance difference with a maximum false discovery rate (FDR) of 0.001 as our criteria for selecting differentially expressed genes and transcripts. We used one-tailed Fisher's exact tests within Blast2GO with a maximum FDR of 0.05 to look for GO term enrichment among the seasonally differentially expressed transcripts relative to the whole suite of transcripts expressed in the ears across both seasons. As described above, assignment of transcripts to KEGG [79] functional pathways was performed based on Blast2GO annotation and used to examine differential expression of these pathways across reproductive states within the SE.

In addition to the differential analysis across the whole transcriptome, we isolated, *in silico*, the normalized read counts of two subsets of transcripts: those that had been annotated as either ion channels or steroid-related. We then performed differential analyses on these subsets with the same methods and parameters used for the whole transcriptome (ie, 4-fold differential abundance and FDR < 0.001). We performed this targeted approach to allow for fewer comparisons and thus a less stringent FDR correction. We selected these particular subsets of transcripts based on previous studies that have implicated steroid hormones and the large conductance, calcium-activated potassium (BK) channel in seasonal variation in auditory sensitivity [12, 13]. This facilitated the identification of more differentially expressed candidate transcripts with important functional implications.

## Availability of supporting data

The final assembled transcriptome and reads from each sample group have been submitted to the NCBI Transcriptome Shotgun Assembly and Sequence Read Archive databases under BioProject accession number [PRJNA269550].

## Additional files

Additional file 1:
**Differentially expressed steroid-related transcripts in saccular epithelium (SE).** Seasonal differential analysis in the SE was performed with the subset of steroid-related transcripts. Top hit BLAST hit descriptions for each transcript are shown. (DOCX 16 kb) Additional file 2:
**Differentially expressed ion channel transcripts in saccular epithelium (SE).** Seasonal differential analysis in the SE was performed with the subset of ion channel transcripts. Top hit BLAST hit descriptions for each transcript are shown. (DOCX 18 kb)

## Abbreviations

BK: Large conductance, calcium-activated potassium
BLAST: Basic local alignment search tool
ERR: Estrogen related receptor
FDR: False discovery rate
FPKM: Fragments per kilobase of transcript per million reads mapped
GO: Gene ontology
KEGG: Kyoto encyclopedia of genes and genomes
SE: Saccular epithelium
VMN: vocal motor nucleus
11KT: 11-ketotestosterone
