# Supplementary material for: Gene expression underlying enhanced, steroid-dependent auditory sensitivity of hair cell epithelium in a vocal fish
Source: BMC Genomics. 2015 Oct 14;16:782. doi: 10.1186/s12864-015-1940-3 (PMC4607102; doi:10.1186/s12864-015-1940-3)
Supplement: Additional file 1: — Differentially expressed steroid-related transcripts in saccular epithelium (SE). Seasonal differential analysis in the SE was performed with the subset of steroid-related transcripts. Top hit BLAST hit descriptions for each transcript are shown. (DOCX 16 kb) [file 12864_2015_1940_MOESM1_ESM.docx]

**Additional file 1.** Differentially expressed steroid related transcripts. Seasonal differential analysis in the SE was performed with the subset of steroid-related transcripts. Top hit BLAST hit descriptions for each transcript are shown.

| **Reproductive Upregulated Transcirpts** |
| --- |
| 17-beta-hydroxysteroid dehydrogenase 14 |
| Cholesterol 25-hydroxylase-like protein member 1 |
| Cytochrome family subfamily a |
| Estrogen-related receptor β type 1 |
| Estrogen-related receptor γ |
| High mobility group protein b2 |
| Hydroxysteroid 11-β-dehydrogenase 1 |
| Lanosterol 14-α demethylase |
| Liver x receptor |
| Non-specific lipid-transfer |
| Non-specific lipid-transfer protein |
| Nuclear receptor ror-α |
| Nuclear receptor subfamily 2 group f member 6 |
| Polyprenol reductase |
| Retinoid x receptor γ |
| Sterol-c4-methyl oxidase |
| Sumo-conjugating enzyme ubc9 |
|  |
| **Non-reproductive Upregulated Transcirpts** |
| Estrogen-related receptor γ |
| Liver x receptor |
| Nuclear receptor subfamily 1 group d member 2 |
| Nuclear receptor subfamily 2 group f member 6 |
| Nuclear receptor subfamily 4 group a member 1 |
| Thyroid hormone receptor α |
