# Supplementary material for: Gene expression underlying enhanced, steroid-dependent auditory sensitivity of hair cell epithelium in a vocal fish
Source: BMC Genomics. 2015 Oct 14;16:782. doi: 10.1186/s12864-015-1940-3 (PMC4607102; doi:10.1186/s12864-015-1940-3)
Supplement: Additional file 2: — Differentially expressed ion channel transcripts in saccular epithelium (SE). Seasonal differential analysis in the SE was performed with the subset of ion channel transcripts. Top hit BLAST hit descriptions for each transcript are shown. (DOCX 18 kb) [file 12864_2015_1940_MOESM2_ESM.docx]

**Additional file 2.** Differentially expressed ion channel transcripts. Seasonal differential analysis in the SE was performed with the subset of ion channel transcripts. Top hit BLAST hit descriptions for each transcript are shown.

| **Reproductive Upregulated Transcripts** |
| --- |
| \| Acetylcholine receptor subunit β \| \| --- \| \| Anoctamin- partial \| \| Anoctamin-10-like isoform x1 \| \| Anoctamin-10-like isoform x4 \| \| ATPase, Ca++ transporting, fast twitch 1 \| \| Calcium-activated potassium channel subunit α-1 \| \| Calcium-activated potassium channel subunit α-1-like isoform 3 \| \| Chloride channel protein 1 \| \| Chloride intracellular channel protein 1 \| \| Chloride intracellular channel protein 2 \| \| Chloride intracellular channel protein 4 \| \| Connexin 30 (Gap junction β-6) \| \| Connexin 36 (Gap junction δ-2) \| \| Connexin 43 (Gap junction α-1) \| \| FXYD domain-containing ion transport regulator 4 \| \| Gamma-aminobutyric acid receptor subunit ρ-1 \| \| Glutamate receptor 4-like isoform 1 \| \| Glycine receptor subunit α-4 \| \| H(+)/Cl(-) exchange transporter 4 isoform 2 \| \| Neuronal acetylcholine receptor subunit α-9-ii \| \| Piezo-type mechanosensitive ion channel component 1 \| \| Potassium sodium hyperpolarization-activated cyclic nucleotide-gated channel 1 \| \| Potassium voltage-gated channel subfamily a member 10 \| \| Potassium voltage-gated channel subfamily a member 2 \| \| Potassium voltage-gated channel subfamily c member 4 \| \| Potassium voltage-gated channel subfamily d member 1 \| \| Potassium voltage-gated channel subfamily h member 7 \| \| Probable glutamate receptor \| \| Ryanodine receptor 2 \| \| Ryanodine receptor 3 \| \| Sodium channel protein type 8 subunit α \| \| Sodium channel subunit β-1 \| \| Sodium channel subunit β-4 \| \| Sodium leak channel non-selective protein isoform 1 \| \| Transient receptor potential cation channel subfamily m member 7 \| \| Trimeric intracellular cation channel type a \| \| Two pore calcium channel protein 1 \| \| Voltage-dependent anion channel 2 \| \| Voltage-dependent anion channel 3 \| \| Voltage-dependent anion-selective channel protein 1 \| \| Voltage-dependent calcium channel subunit α-2 δ-3 \| \| Voltage-dependent P/Q-type calcium channel subunit α-1A \| |
|  |
| **Non-reproductive Upregulated Transcripts** |
| Anoctamin-10-like isoform x4 |
| Anoctamin-4-like isoform x2 |
| Anoctamin-5-like isoform x2 |
| Anoctamin-5-like isoform x4 |
| Chloride intracellular channel protein 1 |
| Chloride intracellular channel protein 4 |
| Connexin 30 (Gap junction β-6) |
| Connexin 43 (Gap junction α-1) |
| Gamma-aminobutyric acid receptor subunit δ |
| Glutamate receptor 2-like isoform 2 |
| Inositol 1,4,5-trisphosphate receptor type 1 |
| Kv channel-interacting protein 4 isoform 5 |
| Piezo-type mechanosensitive ion channel component 1 |
| Potassium voltage-gated channel subfamily a member 2 |
| Potassium voltage-gated channel subfamily kqt member 2 |
| Protein piezo1 |
| Ras GTPase-activating protein 3 |
| Ryanodine receptor 3 |
| Sodium channel subunit β-4 |
| Sodium leak channel non-selective protein isoform 1 |
| Transient receptor potential cation channel subfamily m member 7 |
| Two pore calcium channel protein 1 |
| Voltage-dependent calcium channel subunit α-2 δ-3 |
